# The St. Petersburg School of Medicine for Women

**Published:** 1896-04

**Authors:** 


### The St. Petersburg School of Medicine for Women.

The Russian Government has assigned an annual grant, equivalent to about £10,000, to the Medical School for Women in St. Petersburg. The city undertakes to provide another £2,400, and private munificence has raised an endowment fund of £70,000. Preliminary courses are already being given, but another year must elapse before the school can be housed in its own buildings.—*British Med. Journal.*

